# Personalizing Obesity Treatment: Real-World Comparison of a Very-Low-Calorie Ketogenic Diet Versus a Whole-Food Mediterranean Ketogenic Diet

**DOI:** 10.3390/metabo16040248

**Published:** 2026-04-05

**Authors:** Davide Masi, Maria Letizia Spizzichini, Elena Colonnello, Daniel Vasquez Barahona, Lucio Gnessi, Daniele Gianfrilli, Mikiko Watanabe

**Affiliations:** 1 Department of Experimental Medicine, Section of Medical Pathophysiology, Food Science and Endocrinology, Sapienza University of Rome, 00161 Rome, Italy; 2 Department of Systems Medicine, University of Rome Tor Vergata, 00133 Rome, Italy; 3 UOC of Otorhinolaryngology, Department of Medical Sciences, Giuliano Isontina Medical Institution, Cattinara Hospital, 34149 Trieste, Italy

**Keywords:** obesity, ketogenic diet, Mediterranean ketogenic diet, very-low-calorie ketogenic diet, weight loss, insulin resistance, Mediterranean diet, personalized nutrition, lifestyle intervention

## Abstract

**Highlights:**

**What are the main findings?**

- A whole-food Mediterranean ketogenic diet (MedKD) achieved weight loss comparable to a very-low-calorie ketogenic diet (VLCKD), with approximately 15% reduction in body weight over 3 months.
- Both interventions significantly improved metabolic parameters, including waist circumference and insulin resistance, with similar lipid profile changes and good tolerability.

**What are the implications of the main findings?**

- A Mediterranean-style ketogenic diet based on conventional foods may represent a feasible alternative to formula-based VLCKD programs for obesity treatment.
- Offering different ketogenic strategies may support personalized dietary interventions and improve adherence in clinical obesity management.

**Abstract:**

**Background/Objectives:** Obesity is a chronic, relapsing disease in which lifestyle modification represents the cornerstone of treatment. Among dietary strategies, ketogenic diets can induce rapid weight loss, whereas the Mediterranean diet is associated with established cardiometabolic benefits but typically produces slower weight reduction. Very-low-calorie ketogenic diets (VLCKDs) are effective for weight loss but are often limited by cost, reliance on meal replacements, and reduced long-term feasibility. This study aimed to evaluate whether a whole-food Mediterranean ketogenic diet with moderate caloric restriction (MedKD) could represent a feasible and effective alternative to VLCKD for weight loss and metabolic improvement in adults with obesity. **Methods:** This 3-month prospective, real-world study compared VLCKD and MedKD in adults with obesity attending a clinical nutrition program. The primary outcome was percentage weight loss. Secondary outcomes included changes in waist circumference, waist-to-height ratio, insulin resistance (HOMA-IR), lipid profile, kidney function, and treatment tolerability. Clinical and biochemical parameters were assessed at baseline and after the intervention. Group differences and time-by-group interactions were analyzed to evaluate changes over the study period. **Results:** Sixty-two participants were enrolled, and 55 completed the study (27 VLCKD, 28 MedKD). Baseline characteristics were generally comparable, although the MedKD group had a higher prevalence of diabetes and higher baseline insulin resistance and triglyceride levels. Both dietary interventions resulted in substantial and comparable weight loss (approximately 15% of initial body weight), accompanied by significant reductions in waist circumference and waist-to-height ratio. Insulin resistance improved in both groups, with a greater reduction in HOMA-IR observed in the MedKD group (time × group *p* = 0.031). Serum creatinine decreased in the VLCKD group and slightly increased in the MedKD group (*p* = 0.025). Changes in lipid profile were not significantly different between groups. No severe adverse events were reported. **Conclusions:** A whole-food Mediterranean ketogenic diet with moderate caloric restriction achieved weight loss and metabolic improvements comparable to those observed with VLCKD over three months. These findings suggest that MedKD may represent a feasible alternative to formula-based ketogenic programs, supporting more flexible and personalized dietary strategies in the clinical management of obesity.

## 1. Introduction

Obesity is a complex chronic disease characterized by excessive adiposity that impairs health and increases the risk of type 2 diabetes, hypertension, cardiovascular disease, and other complications [1,2]. Lifestyle modification, particularly diet, remains the cornerstone of treatment, although the optimal dietary approach for weight loss is still debated [3]. Among the most studied strategies are low-carbohydrate ketogenic diets and the Mediterranean diet. Ketogenic diets (KD) have demonstrated efficacy in short-term weight loss and improvements in metabolic parameters, including insulin sensitivity and triglycerides [4,5]. A widely used approach is the very-low-calorie ketogenic diet (VLCKD), which provides a heavily restricted energy intake (typically 600–800 kcal/day) through very low carbohydrates, adequate high-biological-value protein, and minimal fat, via commercial meal replacements (formula soups, shakes, protein bars, etc.). Clinical studies have demonstrated that VLCKDs can lead to significant body weight reductions within a few months and improve glycemic control, blood lipids, and blood pressure, making them a useful short-term therapy in patients with obesity. VLCKDs have been included in medical practice and guidelines, provided they are medically supervised [6]. However, reliance on commercial meal products in VLCKD protocols can be costly and to some patients less appealing [7]. Moreover, despite some overlapping elements with the Mediterranean diet (MedD) (e.g., emphasis on olive oil), traditional VLCKDs do not usually include many low-carbohydrate staples of the MedD such as fish, seeds, nuts, and red wine. By contrast, the MedD is a primarily plant-based, moderately high-carbohydrate diet rich in fruits, vegetables, whole grains, legumes, olive oil, and fish, and associated with cardiovascular and metabolic benefits in the long term [8,9]. While the MedD is linked to weight stability and health, when applied with only modest caloric restriction it tends to produce relatively small weight loss compared to other regimens [10]. Over time, adherence rather than diet type appears to be the key determinant of success [11]. Therefore, personalization is key, and finding more effective approaches, blending existing ones and further adjusting to the single patient, is the key to success. The concept of blending the two dietary options is not new: in recent years, there has been growing interest in Mediterranean-style ketogenic diets as a potential compromise that leverages the advantages of both approaches. A Mediterranean ketogenic diet (MedKD) applies the carbohydrate restriction of a classical ketogenic diet while encouraging foods typical of the Mediterranean tradition: non-starchy vegetables, olive oil and nuts as primary fats, moderate amounts of fish and poultry as protein sources, eggs, and herbs/spices for flavoring. In some versions, moderate red wine intake is permitted and dairy and red meat are limited, aligning with Mediterranean dietary principles. Early studies have shown the feasibility and benefits of such diets. For example, Pérez-Guisado et al. first reported in their one-arm pilot study on a “Spanish ketogenic Mediterranean diet” without calorie restriction, which included olive oil, fish, and red wine; over 12 weeks, participants lost ~14 kg and saw improvements in blood pressure, glucose, and triglycerides [12]. Similarly, Paoli et al. tested a ketogenic Mediterranean diet with phytoextracts (KEMEPHY) of 1000–1200 kcal/day alternating with periods of a standard MedD, and observed ~7.8% body weight reduction in 6 weeks alongside improvements in cardiovascular risk markers [13]. More recently, a randomized study in patients with type 2 diabetes or prediabetes compared a very-low-calorie ketogenic Mediterranean diet to a standard hypocaloric Mediterranean diet: the keto-Med arm led to greater reductions in body weight, fasting glucose, glycated hemoglobin, insulin, and triglycerides [14]. To date, evidence remains limited and long-term sustainability is unclear. Furthermore, no direct comparisons between the conventional meal-replacement VLCKD and a whole-food MedKD approach have been made. In clinical practice, allowing patients to choose a diet that suits their food preferences and lifestyle could improve adherence, which is crucial for success [11]. We hypothesized that a MedKD without extreme caloric restriction or meal replacements could achieve weight loss and metabolic benefits comparable to a VLCKD, while offering greater flexibility and cultural integration. Therefore, the aim of this real-world study was to compare the efficacy and safety of a commercial VLCKD versus a whole-food MedKD in patients with obesity over a 3-month period.

## 2. Materials and Methods

### 2.1. Study Design and Participants

We conducted a 3-month real-world comparative intervention study in adults with obesity. This study was designed as a prospective, non-randomized trial in which participants selected one of two dietary interventions based on personal preference. The choice of a non-randomized, preference-based allocation was intended to reflect real-world clinical practice, where dietary adherence is strongly influenced by patient preference. This design allows evaluation of effectiveness under routine care conditions but may introduce selection bias. Eligibility criteria included age ≥ 18 years and a body mass index (BMI) ≥ 30 kg/m^2^. Patients were excluded if they had any contraindications to ketogenic diets (such as advanced kidney or liver disease, pregnancy or lactation, insulin dependent diabetes) [15]. All participants provided informed consent and received detailed information about both diet options before choosing their preferred intervention. The study protocol was approved by the local Ethics Committee (ref. 5475, approved 6 May 2024), and all participants provided written informed consent. The study was conducted in accordance with the Declaration of Helsinki.

### 2.2. Dietary Interventions

Patients in the VLCKD group followed a strict ketogenic diet plan using commercial meal replacements as the primary source of nutrition. The VLCKD program provided approximately 600–800 kcal per day, divided into 4–5 meal-replacement products (shakes, soups, bars, etc.) plus low-carbohydrate vegetables. The macronutrient composition was carbohydrate ≤ 50 g/day, protein ~1.2–1.5 g/kg ideal body weight, and a limited amount of fat, to come primarily from extra-virgin olive oil. Patients were instructed to supplement with at least 2 L of water per day and a multivitamin and mineral preparation (including sodium, potassium, magnesium) to prevent electrolyte imbalances. Patients had regular check-ins to monitor compliance and manage any side effects. Patients in the MedKD group followed a whole-food ketogenic diet modeled on Mediterranean dietary principles, with no meal replacement products. Instead, patients consumed conventional foods with a moderate energy restriction typically yielding 1200–1500 kcal/day, adjusted to individual factors such as sex and baseline weight. The diet was formulated to keep carbohydrates < 50 g per day to induce ketosis, while deriving proteins and fats from Mediterranean-favored sources. Emphasis was placed on generous intake of non-starchy vegetables and extra-virgin olive oil as the main added fat. Nuts and seeds (e.g., almonds, walnuts, pumpkin seeds) were recommended in moderation as snacks or salad additions. Protein was provided through fish and seafood (at least 2–3 times per week), poultry, and eggs, with limited lean red meat. Moderate portions of cheese or yogurt were allowed. To maintain Mediterranean flavor and variety under carb restriction, participants were encouraged to use herbs, spices, garlic, and lemon. Low-sugar fruits (such as berries) could be consumed in small quantities occasionally, but starchy foods (bread, pasta, rice, potatoes) and sugary foods were eliminated. Daily vegetable intake ranged between approximately 400–600 g, primarily from non-starchy sources, to ensure micronutrient adequacy and support satiety. Depending on individual tolerance, ~1 glass (~150 mL) of red wine was permitted with meals once or twice per week as the only alcoholic beverage, though this was optional. A representative 7-day meal plan for the Mediterranean ketogenic diet (MedKD) is provided in Supplementary Table S1. All participants were educated on ketosis and its potential side effects at the start. They were given meal plans and recipes tailored to their assigned diet. The importance of maintaining hydration and adequate electrolyte intake was stressed. Both groups were asked to engage in at least light-to-moderate physical activity (e.g., regular walking) as tolerated, and physical activity levels were recorded. Adherence was encouraged through biweekly phone or in-person consultations where dietitians monitored weight, checked for urinary ketones (assessed using semi-quantitative dipstick tests), and reinforced dietary guidelines. However, no standardized quantitative adherence score was applied.

### 2.3. Assessments

Clinical and laboratory assessments were conducted at baseline (week 0) and at 3 months (week 12). At each visit, anthropometric measurements were taken with participants in light clothing and no shoes. Body weight was measured to the nearest 0.1 kg using a calibrated scale. Height was recorded at baseline using a stadiometer, and BMI was calculated as kg/m^2^. Waist circumference was measured at the midpoint between the lowest rib and the iliac crest. We also computed the waist-to-height ratio (WHtR) as an index of central adiposity. Fasting venous blood samples were collected in the morning (after an overnight fast of ≥8 h) at baseline and 3 months. Insulin resistance was estimated using the Homeostasis Model Assessment index (HOMA-IR), calculated as fasting insulin (μU/mL) × fasting glucose (mmol/L)/22.5. The estimated glomerular filtration rate (eGFR) was calculated from serum creatinine using the CKD-EPI equation, to evaluate renal function. Participants were interviewed about diet tolerability and side effects at the 3-month visit. They were asked to report any symptoms such as excessive fatigue, dizziness, constipation, nausea, or others.

### 2.4. Outcomes and Statistical Analysis

The primary outcome was the change in body weight from baseline to 3 months. Secondary outcomes included changes in BMI, waist circumference, and metabolic parameters (glycemic indices, HOMA-IR, lipid profile, uric acid, etc.), as well as measures of safety (electrolytes, liver and kidney function) and tolerability (incidence of side effects). Data are presented as mean ± standard deviation for continuous variables or as number (percentages) for categorical variables. Non-normally distributed variables were log-transformed. Between-group comparisons of baseline characteristics were performed using independent-samples *t* tests for continuous variables and the χ^2^ or Fisher’s exact tests for categorical variables, as appropriate. Within-group changes from baseline were evaluated with paired *t* tests. A general linear repeated measures model, with terms for treatment, time, time*treatment interaction, was used to analyze continuous endpoints. The time*treatment interaction analyses were used to determine groupwise differences. Although this approach accounts for within-subject changes over time, no additional covariate adjustment (e.g., inclusion of baseline values or metabolic variables as covariates) was performed. Therefore, residual confounding due to baseline imbalances cannot be excluded. The sample size calculation, based on an expected standard deviation of approximately 7% in weight loss, indicated that a total of 50 participants was sufficient to demonstrate non-inferiority of the MedKD compared to the VLCKD, with a non-inferiority margin of 4%, 80% power, and a one-sided alpha of 0.025. Anticipating a potential 20% drop-out rate, 62 patients were enrolled. Analyses were performed IBM SPSS Statistics for Windows, version 27.0 (IBM Corp., Armonk, NY, USA), available through Sapienza University of Rome institutional license. All statistical tests were two-tailed, and a *p*-value < 0.05 was considered statistically significant.

## 3. Results

A total of 62 participants were enrolled, and 55 completed the 3-month study (27 VLCKD, 28 MedKD). Baseline characteristics are summarized in Table 1. The two groups were comparable for age, sex distribution, BMI, and most comorbidities. However, MedKD participants had a higher prevalence of diabetes (4 vs. 0; *p* = 0.041), higher triglycerides (143.9 ± 69.9 vs. 107.7 ± 45.1 mg/dL; *p* = 0.026), and greater insulin resistance (HOMA-IR 6.5 ± 4.6 vs. 2.94 ± 1.7; *p* = 0.003) at baseline compared with the VLCKD group. The prevalence of hypertension, dyslipidemia, and other metabolic comorbidities did not differ significantly (Table 1).

Both diets produced marked and comparable reductions in body weight and BMI, with a mean weight loss approaching 15% of initial body weight in each group. Waist circumference and waist-to-height ratio also decreased significantly within both groups, with no differential effect between them (time × group *p* > 0.8).

Fasting glucose declined significantly in both groups, with a trend toward a larger absolute decrease in MedKD than in VLCKD (*p* = 0.068). HOMA-IR improved substantially in both groups, showing a greater reduction in MedKD than in VLCKD (time × group *p* = 0.031). HbA1c decreased modestly and did not differ between groups (time × group *p* = 0.205) (Table 1).

Lipid changes were directionally favorable in both interventions. LDL-cholesterol declined (time × group *p* = 0.163), and HDL-cholesterol only decreased slightly in VLCKD while remaining stable in MedKD (time × group *p* = 0.056). Triglycerides dropped significantly within both groups, with a trend toward a larger improvement in MedKD (time × group *p* = 0.064), consistent with their higher baseline values (Table 1).

Serum creatinine decreased in the VLCKD group and slightly increased in the MedKD group, yielding a significant time × group interaction (*p* = 0.025). Despite the statistical difference, absolute changes were small and remained within the physiological range. Liver transaminases (AST, ALT) and electrolytes (sodium, potassium) showed significant within-group improvements but no between-group differences (Table 1).

Self-reported physical activity increased in both groups, with a trend toward greater increase in MedKD (time × group *p* = 0.072). Both diets were well tolerated, with no serious adverse events reported. Minor gastrointestinal symptoms were mild and transient; their prevalence did not differ significantly between groups. A non-significant trend toward higher post-diet bloating was observed in MedKD (time × group *p* = 0.080) (Table 1).

Data are expressed as mean ± standard deviation (SD) for continuous variables and as number (percentage) for categorical variables. p_1_ indicates the between-group comparison at baseline, performed using independent-samples *t* tests for continuous variables and χ^2^ or Fisher’s exact tests for categorical variables. p_2_ indicates the time × group interaction derived from the general linear repeated-measures model, reflecting differential change over time between VLCKD and MedKD. A *p* value < 0.05 was considered statistically significant. Abbreviations: VLCKD, very-low-calorie ketogenic diet; MedKD, Mediterranean ketogenic diet; BMI, body mass index; HOMA-IR, homeostatic model assessment of insulin resistance; HbA1c, glycated hemoglobin.

## 4. Discussion

In this real-world comparative study, we evaluated two distinct ketogenic approaches for obesity: a formula-based VLCKD and a whole-food MedKD with moderate caloric restriction. After three months, both interventions achieved substantial and clinically meaningful weight loss of approximately 15% of initial body weight, with no difference between groups. These results indicate that a ketogenic regimen based on Mediterranean food choices can match the short-term efficacy of the more restrictive VLCKD, offering a culturally integrated and sustainable alternative.

A key methodological consideration of this study is the non-randomized design, in which participants selected their preferred dietary intervention. This approach reflects real-world clinical practice but introduces the potential for selection bias and limits internal validity. Indeed, several baseline differences were observed between groups, including a higher prevalence of diabetes, greater insulin resistance, and higher triglyceride levels in the MedKD group, as well as a trend toward different sex distribution. These imbalances may have influenced the magnitude of metabolic responses, particularly the greater improvement in HOMA-IR observed in the MedKD group, which may partly reflect regression toward the mean or greater baseline disease severity. Therefore, the present findings should be interpreted cautiously as exploratory and hypothesis-generating, rather than establishing causal equivalence between interventions.

The ~15 kg average weight loss observed in both groups over 12 weeks is striking and clinically meaningful. It corresponds to 14–15% of body weight, which exceeds the typical 5–10% weight loss often seen with traditional calorie-focused diets in similar time frames. Achieving >15% weight loss is noteworthy because it is above the threshold typically associated with remission of type 2 diabetes and major metabolic improvements [3]. Indeed, several of our patients with diabetes or prediabetes experienced normalization of glucose and HbA1c, underscoring the therapeutic potential of these diets beyond weight loss alone. The comparable weight loss between VLCKD and MedKD suggests that severe energy restriction is not the only route to major weight reduction—even with moderate caloric deficits, if the diet induces ketosis, robust appetite suppression and increased fat oxidation may yield net caloric deficit similar to that of much stricter protocols [13]. Importantly, the observation that two ketogenic regimens with markedly different prescribed caloric intakes produced nearly identical weight loss is not unexpected and is supported by existing literature. Under conditions of nutritional ketosis, actual caloric intake often converges independently of the prescribed targets due to the strong satiety effect of low-carbohydrate diets. In the randomized trial by Brehm et al., women following an ad libitum very-low-carbohydrate ketogenic diet spontaneously reduced their caloric intake to levels comparable to those assigned to a calorie-restricted low-fat diet, yet achieved significantly greater weight loss [16]. This demonstrates that ketogenic diets can naturally induce an energy deficit even without formal caloric restriction, and that apparent differences in prescribed calories do not necessarily translate into meaningful differences in effective energy intake.

Furthermore, weight regulation cannot be fully explained by prescribed calories alone. Several physiological mechanisms, including differential substrate oxidation, thermic effect of food, adaptive thermogenesis, and interindividual variability in hormonal responses, can lead to different weight-loss outcomes despite similar caloric intake [17]. Moreover, the landmark work by Leibel, Rosenbaum, and Hirsch demonstrated substantial interindividual variability in metabolic adaptation, indicating that equal caloric deficits do not necessarily yield equal weight loss [18]. Taken together, these findings support the plausibility of our results: in a ketogenic context, both the real caloric deficit and the weight-loss response depend on complex metabolic adjustments rather than on caloric prescription alone.

Moreover, an important and relatively underexplored aspect is the role of food quality within ketogenic dietary patterns. While both interventions achieved similar macronutrient targets, the MedKD emphasized whole, minimally processed foods characteristic of the Mediterranean diet, whereas the VLCKD relied heavily on meal replacements. Emerging evidence indicates that dietary processing independently influences energy intake and metabolic health. In a randomized controlled trial, ultra-processed diets led to increased caloric intake and weight gain compared to unprocessed diets [19]. In this context, the MedKD may offer advantages related to satiety, dietary satisfaction, and metabolic regulation, potentially mediated by higher fiber content, micronutrient density, and beneficial effects on the gut microbiota and inflammatory pathways.

Beyond weight loss, both diets improved several metabolic parameters. Glycemic indices and triglycerides decreased in parallel with adiposity, while HDL-C and LDL-C remained stable, and no significant between-group differences were observed for any lipid parameter. HOMA-IR improved significantly in both groups, with a greater reduction in MedKD. However, this result must be interpreted cautiously given the higher baseline HOMA-IR and prevalence of diabetes in the MedKD group, which provided greater potential for improvement. Fasting glucose and HbA1c changes were similar between interventions, suggesting that once ketosis and weight loss are achieved, both protocols confer comparable short-term glycemic benefits. The greater reduction in HOMA-IR observed in the MedKD group warrants cautious interpretation given baseline differences; however, it may also reflect physiological mechanisms linked to dietary composition. The Mediterranean dietary pattern is rich in monounsaturated fatty acids, particularly from extra-virgin olive oil, which have been associated with improved insulin sensitivity and reduced inflammatory signaling [3]. Additionally, adherence to a Mediterranean diet has been shown to improve glucose metabolism and insulin action independently of weight loss [13], suggesting a potential additive benefit beyond carbohydrate restriction alone.

Serum creatinine decreased modestly in VLCKD and slightly increased in MedKD, although all values remained within the normal range. These small differences indicate divergent but clinically insignificant renal trajectories, supporting the short-term safety of both regimens under medical supervision. Our results echo those of recent studies that found no adverse effect of ketogenic weight-loss diets on kidney function in patients without chronic kidney disease [20]. These divergent trends may reflect differences in hydration status, dietary protein intake, or subtle changes in muscle mass between interventions, although the absence of body composition data prevents definitive interpretation.

Importantly, both interventions were well tolerated. No serious adverse events occurred, and the frequency of minor MedKD diet likely enhanced adherence, mirroring clinical practice where individual preference strongly influences success. From a behavioral standpoint, the whole-food MedKD may facilitate transition to long-term maintenance, as it already incorporates culturally familiar foods and avoids the discontinuity associated with reintroducing normal meals after a formula-based VLCKD.

Several limitations should be acknowledged. The non-randomized design allows for self-selection bias, and the short duration precludes assessment of long-term weight maintenance and cardiometabolic outcomes. In fact, the study included only two assessment time points (baseline and 3 months), which precludes characterization of the temporal trajectory of weight loss, the timing of metabolic adaptations, and adherence dynamics over the course of the intervention. Additionally, our sample size provides reasonable power for large effects, but smaller differences might not have been detected. Nevertheless, the magnitude and consistency of the observed effects strengthen the evidence that both ketogenic strategies are potent short-term interventions for obesity. Moreover, the study was conducted in a single center with only Caucasian European patients, which may limit generalizability to other populations and cultural contexts. However, the concept of a MedKD could be adapted to various cuisines (e.g., using local sources of healthy fats and proteins) around the world. An additional limitation is that we did not directly assess patients’ actual food intake during the intervention. Although both diets were delivered with clear caloric targets, we did not collect quantitative dietary records or use objective tools to estimate real caloric consumption. This prevents us from determining whether the effective energy intake differed between groups or converged over time, as frequently observed in ketogenic settings due to ketosis-induced satiety. As a result, we cannot quantify the extent to which the comparable weight loss observed across interventions was driven by similar spontaneous energy intake reductions rather than by the prescribed caloric frameworks. Finally, body composition was not assessed, preventing differentiation between fat mass and lean mass changes and limiting interpretation of the quality of weight loss. Although waist circumference was used as a surrogate marker of central adiposity, it does not fully capture changes in body composition. In addition, capillary ketone levels were not measured due to the real-world setting; however, urinary acetoacetate dipsticks were used as acceptable proxies for nutritional ketosis.

An additional limitation relates to baseline imbalances between groups, including differences in diabetes prevalence, triglycerides, and insulin resistance. Although the repeated-measures model captures within-subject changes over time, no additional covariate-adjusted analyses (e.g., ANCOVA including baseline values) or propensity score methods were performed. Therefore, residual confounding cannot be excluded, and the observed between-group differences—particularly for HOMA-IR—should be interpreted with caution.

A further limitation is the absence of inflammatory markers. Obesity is characterized by chronic low-grade inflammation, and indices derived from hematological parameters—such as the neutrophil-to-lymphocyte ratio (NLR), systemic inflammatory response index (SIRI), and related biomarkers—as well as C-reactive protein, may provide additional insight into cardiometabolic risk. These parameters were not systematically collected in the present real-world setting. Nevertheless, the consistent improvements observed across key metabolic parameters—including insulin resistance, triglycerides, and central adiposity—support a favorable shift in cardiometabolic risk, which is typically associated with reduced systemic inflammation.

Future studies should adopt more robust methodological approaches, including randomized controlled designs, stratified randomization (e.g., by diabetes status), or preference-based trial designs. In addition, the incorporation of inflammatory indices would help elucidate the mechanistic effects of ketogenic dietary interventions, while the use of covariate-adjusted models or propensity score matching/weighting would improve comparability between groups and strengthen causal inference. Future randomized controlled trials with larger sample sizes and longer follow-up, incorporating structured dietary records, digital tracking tools, or objective measures of energy intake, will be essential to better elucidate the relationship between prescribed diets, actual intake, and metabolic outcomes, necessary to confirm the long-term effectiveness and sustainability of MedKD.

## 5. Conclusions

In this real-world comparative study, both a very-low-calorie ketogenic diet based on meal replacements and a whole-food MedKD produced substantial and comparable improvements in weight and metabolic health over three months. These findings reinforce the concept that personalization is central to dietary obesity management. Different ketogenic strategies may serve distinct patient needs—formula-based VLCKD programs can offer structure and rapid implementation, whereas whole-food MedKD plans may enhance flexibility, cultural fit, and long-term adherence. Rather than competing approaches, they represent complementary tools within the therapeutic arsenal, allowing clinicians to tailor treatment to individual preferences, metabolic profiles, and clinical contexts.

## Figures and Tables

**Table 1:** Patients baseline characteristics and changes over time.

	Baseline		3 Months			
Parameter (Unit of Measure)	VLCKD (Mean ± SD or n (%))	MedKD (Mean ± SD or n (%))	VLCKD (Mean ± SD or n (%))	MedKD (Mean ± SD or n (%))	p1	p2
Clinical and demographic characteristics						
Age (years)	49.59 ± 8.997	46.46 ± 15.133			0.354	
Female sex, n	21 (77.8)	15 (53.6)			0.059	
Diabetes, n	0 (0.0)	4 (14.3)			0.041	
Hypertension, n	5 (18.5)	8 (28.6)			0.38	
Dyslipidemia, n	9 (33.3)	15 (53.6)			0.13	
Antihypertensive therapy, n	6 (22.2)	6 (21.4)	3 (11.1)	5 (17.9)	0.477	0.478
Regular physical activity, n	6 (22.2)	8 (28.6)	8 (29.6)	15 (53.6)	0.589	0.072
Anthropometric parameters						
BMI (kg/m^2^)	36.92 ± 5.6	37.55 ± 6.1	31.36 ± 4.6	32.20 ± 5.9	0.695	0.741
Body weight (kg)	99.44 ± 21.2	108.59 ± 18.9	84.29 ± 17.3	93.15 ± 18.1	0.098	0.88
Waist circumference (cm)	111.04 ± 15.5	115.98 ± 12.8	97.81 ± 14	101.83 ± 12.7	0.209	0.835
Waist-to-height ratio	1.4939 ± 0.16749	1.4804 ± 0.17462	1.6999 ± 0.20362	1.6903 ± 0.20888	0.774	0.967
Biochemical and metabolic parameters						
Fasting glucose (mg/dL)	95.85 ± 9.155	102.07 ± 28.820	93.33 ± 8.963	89.78 ± 11.152	0.286	0.068
Creatinine (mg/dL)	0.875 ± 0.1659	0.868 ± 0.1833	0.782 ± 0.1196	0.892 ± 0.1785	0.885	0.025
Sodium (mmol/L)	140.54 ± 2.043	140.04 ± 2.619	140.46 ± 2.502	140.04 ± 2.391	0.473	0.988
Potassium (mmol/L)	4.429 ± 0.3954	4.325 ± 0.4671	4.657 ± 0.4258	4.367 ± 0.4156	0.414	0.214
AST (U/L)	21.32 ± 7.559	27.56 ± 21.938	18.65 ± 4.079	21.08 ± 7.161	0.189	0.256
ALT (U/L)	27.52 ± 20.953	32.24 ± 22.378	18.62 ± 5.162	24.80 ± 12.309	0.445	0.752
Total cholesterol (mg/dL)	201.07 ± 30.466	195.46 ± 37.673	182.16 ± 29.100	183.53 ± 52.670	0.546	0.213
LDL cholesterol (mg/dL)	129.70 ± 35.1093	115.16 ± 38.1124	117.08 ± 32.2	113.61 ± 51.5	0.147	0.163
HDL cholesterol (mg/dL)	53.96 ± 16.2	51.52 ± 11.1	47.95 ± 12.090	50.88 ± 10.612	0.532	0.056
Triglycerides (mg/dL)	107.67 ± 45.076	143.93 ± 69.860	85.63 ± 33.083	93.33 ± 36.064	0.026	0.064
Uric acid (mg/dL)	5.285 ± 1.2	5.985 ± 1.2	5.010 ± 1.2	5.874 ± 1.2	0.072	0.783
Insulin (µU/mL)	12.08 ± 6.9	23.58 ± 11.1	11.98 ± 14.4	17.85 ± 12.9	<0.001	0.098
HOMA-IR	2.941 ± 1.7	6.5 ± 4.6	2.655 ± 2.8	4.273 ± 3.2	0.003	0.031
HbA1c (%)	5.546 ± 0.3713	5.989 ± 1.3	5.394 ± 0.2401	5.401 ± 0.5577	0.178	0.205
Symptoms and tolerability						
Reflux symptoms, n	5 (18.5)	4 (14.3)	1 (3.7)	0 (0.0)	0.671	0.304
Heartburn, n	4 (14.8)	0 (0.0)	0 (0.0)	0 (0.0)	0.034	1
Flatulence, n	1 (3.7)	1 (3.6)	0 (0.0)	2 (7.1)	0.979	0.157
Bloating, n	2 (7.4)	4 (14.3)	0 (0.0)	3 (10.7)	0.413	0.08
Abdominal pain, n	2 (7.4)	1 (3.6)	0 (0.0)	0 (0.0)	0.531	1
Constipation, n	5 (18.5)	5 (17.9)	7 (25.9)	12 (42.9)	0.949	0.187
Diarrhea, n	3 (11.1)	4 (14.3)	0 (0.0)	1 (3.6)	0.724	0.322
Muscle weakness, n	2 (7.4)	0 (0.0)	1 (3.7)	2 (7.1)	0.142	0.574
Presyncope, n	0 (0.0)	0 (0.0)	0 (0.0)	1 (3.6)	1	0.322
Palpitations, n	2 (7.4)	1 (3.6)	0 (0.0)	0 (0.0)	0.531	1
Mental fatigue, n	4 (14.8)	2 (7.1)	1 (3.7)	3 (10.7)	0.362	0.317

## Data Availability

The data sets generated and/or analyzed during the current study are not publicly available but are available from the corresponding author on reasonable request.

## Supplementary Materials

The following supporting information can be downloaded at: https://www.mdpi.com/article/10.3390/metabo16040248/s1, Table S1: Example 7-day MedKD meal plan.

## Abbreviations

The following abbreviations are used in this manuscript:

VLCKD	Very-Low-Calorie Ketogenic Diet
MedKD	Mediterranean Ketogenic Diet
KD	Ketogenic Diet
MedD	Mediterranean Diet
BMI	Body Mass Index
WHtR	Waist-to-Height Ratio
HOMA-IR	Homeostasis Model Assessment of Insulin Resistance
HbA1c	Glycated Hemoglobin
AST	Aspartate Aminotransferase
ALT	Alanine Aminotransferase
eGFR	Estimated Glomerular Filtration Rate
SD	Standard Deviation
