# Supplementary material for: Personalizing Obesity Treatment: Real-World Comparison of a Very-Low-Calorie Ketogenic Diet Versus a Whole-Food Mediterranean Ketogenic Diet
Source: Metabolites. 2026 Apr 5;16(4):248. doi: 10.3390/metabo16040248 (PMC13117268; doi:10.3390/metabo16040248)
Supplement: Supplementary file 1 [file metabolites-16-00248-s001.zip › metabolites-4238853-supplementary.pdf]

Table S1. Example 7-day MedKD meal plan.

Day	Breakfast	Lunch	Dinner	Snacks
Day 1	Greek yogurt (150 g) + walnuts (20 g) + chia seeds (10 g)	Grilled chicken (150 g) with salad: mixed greens (100 g), cucumber (100 g), olives (30 g), olive oil (10 g)	Salmon (150 g) with zucchini (150 g) and eggplant (150 g), olive oil (10 g)	Almonds (20 g)
Day 2	Omelet (2 eggs) with spinach (100 g), feta (40 g), olive oil (10 g)	Tuna (120 g) with lettuce (100 g), cherry tomatoes (80 g), capers (10 g), olive oil (10 g)	Turkey breast (150 g) with broccoli (200 g), olive oil (10 g)	Greek yogurt (100 g) + hazelnuts (15 g)
Day 3	Ricotta (100 g) + flaxseeds (10 g)	Eggplant parmigiana: eggplant (250 g), tomato sauce (80 g), olive oil (10 g), cheese (40 g)	Sea bass (150 g) with asparagus (200 g), olive oil (10 g)	Olives (30 g) + walnuts (15 g)
Day 4	Scrambled eggs (2) with avocado (80 g), olive oil (10 g)	Chicken (150 g) with arugula (100 g), zucchini (100 g), parmesan (30 g), olive oil (10 g)	Beef (150 g) with spinach (150 g) and mushrooms (150 g), olive oil (10 g)	Greek yogurt (100 g) + almonds (15 g)
Day 5	Greek yogurt (150 g) + pumpkin seeds (15 g) + berries (50 g)	Salmon (150 g) with salad: lettuce (80 g), cucumber (100 g), zucchini (100 g), olive oil (10 g)	Chicken thighs (150 g) with zucchini (150 g) and bell peppers (100 g), olive oil (10 g)	Hazelnuts (20 g)
Day 6	Omelet (2 eggs) with mushrooms (100 g), goat cheese (40 g), olive oil (10 g)	Sardines (120 g) with salad: lettuce (100 g), cucumber (100 g), olive oil (10 g)	Turkey meatballs (150 g) with eggplant (200 g) and tomato sauce (80 g), olive oil (10 g)	Olives (30 g) + walnuts (15 g)
Day 7	Ricotta (100 g) + almonds (15 g) + cocoa (5 g, unsweetened)	Shrimp (150 g) with avocado (80 g), lettuce (100 g), zucchini (100 g), olive oil (10 g)	Lamb (150 g) with zucchini (150 g) and eggplant (100 g), olive oil (10 g)	Greek yogurt (100 g), mixed nuts (15 g)

Carbohydrate intake was maintained below 50 g/day to promote nutritional ketosis. Vegetable quantities refer to raw weight and were selected to ensure adequate fiber and micronutrient intake while maintaining low glycemic load. Daily vegetable intake ranged between approximately 400 and 600 g, primarily from non-starchy sources (e.g., leafy greens, zucchini, eggplant, broccoli, asparagus). Portion sizes were individualized in clinical practice, but this template reflects typical prescriptions used in the study setting.
